# Subpopulation augmentation among habitat patches as a tool to manage an endangered Mojave Desert wetlands-dependent rodent during anthropogenic restricted water climate regimes

**DOI:** 10.1371/journal.pone.0224246

**Published:** 2019-10-24

**Authors:** Andrés M. López-Pérez, Janet Foley, Austin Roy, Risa Pesapane, Stephanie Castle, Amanda Poulsen, Deana L. Clifford

**Affiliations:** 1 Department of Medicine and Epidemiology, School of Veterinary Medicine, University of California, Davis, California, United States of America; 2 Wildlife Investigations Lab, California Department of Fish and Wildlife, Rancho Cordova, California, United States of America; Museu de Ciències Naturals de Granollers, SPAIN

## Abstract

Intensive management may be necessary to protect some highly vulnerable endangered species, particularly those dependent on water availability regimes that might be disrupted by ongoing climate change. The Amargosa vole (*Microtus californicus scirpensis*) is an increasingly imperiled rodent constrained to rare wetland habitat in the Mojave Desert. In 2014 and 2016, we trapped and radio-collared 30 voles, 24 were translocated and six remained at donor and recipient marshes as resident control voles. Soft-release was performed followed by remote camera and radio-telemetry monitoring. Although comparative metrics were not statistically significant, the mean maximum known distance moved (MDM) was longer for translocated (82.3 ± 14.6 m) vs. resident-control voles (74.9 ± 17.5 m) and for female (98.4 ± 19.9 m) vs. male (57.8 ± 9.1 m) voles. The mean area occupied (AO) tended to be greater in female (0.15 ± 0.04 ha) vs. male (0.12 ± 0.03 ha) voles, and control voles (0.15 ± 0.05 ha) compared with translocated voles (0.13 ± 0.03 ha). The mean minimum known time alive (MTA) was 38.2 ± 19.4 days for resident-control voles and 47.0 ± 10.6 days for translocated voles. Female survival (55.7 ± 14.3 days) exceeded that of males (31.5 ± 9.4 days) regardless of study group. Activity in bulrush/rushes mix and bulrush vegetation types was strongly and significantly overrepresented compared with salt grass and rushes alone, and habitat selection did not differ between resident and translocated voles. Our results provide ecological and methodological insights for future translocations as part of a strategy of promoting long-term survival of an extremely endangered small mammal in a wild desert environment.

## Introduction

Over the last decades, earth has experienced a human-driven wave of high extinction rates and global loss of biodiversity caused by climate change and habitat loss and fragmentation [1, 2]. As species and populations decrease, and wildlife-human conflict becomes more frequent, human-mediated movement of wild animals (translocation or reintroduction) may be a valuable tool for wildlife conservation and management programs [3]. In fact, species translocations are increasing worldwide in several taxonomic groups including birds [4] and various mammals [5, 6]. Translocation is most likely to succeed when informed by data regarding natural activity, habitat requirements, space-use and movement patterns, and behavior of the animals being translocated [7–9].

The Amargosa vole, *Microtus californicus* subsp. *scirpensis*, is an endangered rodent endemic to the Tecopa region of the Mojave Desert of California [10]. The vole is obligately dependent on wetlands near the Amargosa River dominated by three-square bulrush (*Schoenoplectus americanus*) [11, 12]. This region of the Mojave Desert is among the most arid of North America [13] and supports numerous rare and endemic species in increasingly isolated wetland habitat patches. Endangered species management in this region must contend with anthropogenic hydrologic alterations (e.g. groundwater pumping and land clearing) and climate changes that often manifest locally as reduced and less reliable available water [14, 15]. With ongoing habitat degradation and loss of available water, the population status of the Amargosa vole is now dire. Large-scale habitat reconstruction for voles is insupportable with available water resources, but small-scale marsh reconstruction projects may be feasible [16], as well as reintroduction of voles into unoccupied but suitable marshes or those presently housing unsustainably small subpopulations [10]. However, translocation of rodents faces particular difficulties, especially one with highly specific habitat requirements and which is a target prey item for abundant local predators [17]. In general, translocated rodents must be able to rapidly find protection from predators, cope with intra-guild competition, and tolerate local pathogens [18]. With adequate data and planning, translocation has been successful in rodents including Heermann’s kangaroo rats (*Dipodomys heermanni*) [6] and the Stephens’ kangaroo rat (*Dipodomys stephensi*) [19].

Our broad goal in this project was to perform population augmentation into sparsely inhabited marshes to reinforce these subpopulations and obtain vitally needed data regarding vole movement, habitat use, and survival upon translocation. The specific goals of this study were to use radio-collars on Amargosa voles, to determine space use, movement patterns and habitat use preferences of translocated voles, and whether predation-related mortality, emigration, or other outcomes might limit success of future larger-scale translocations into rehabilitated habitat. We tested this by translocating individuals to new marshes, comparing outcomes to voles in donor and recipient marsh controls.

## Material and methods

### Study area

Work was performed in accordance with the guidelines of the American Society of Mammalogists [20], US Fish and Wildlife Service (USFWS) and California Department of Fish & Wildlife (CDFW) Memoranda of Understanding, US Fish and Wildlife Service Amargosa vole Recovery Permit #TE546414A-2, a permit from Bureau of Land Management (BLM), and approval by the UC Davis Institutional Animal Care and Use Committee.

**Fig 1:**
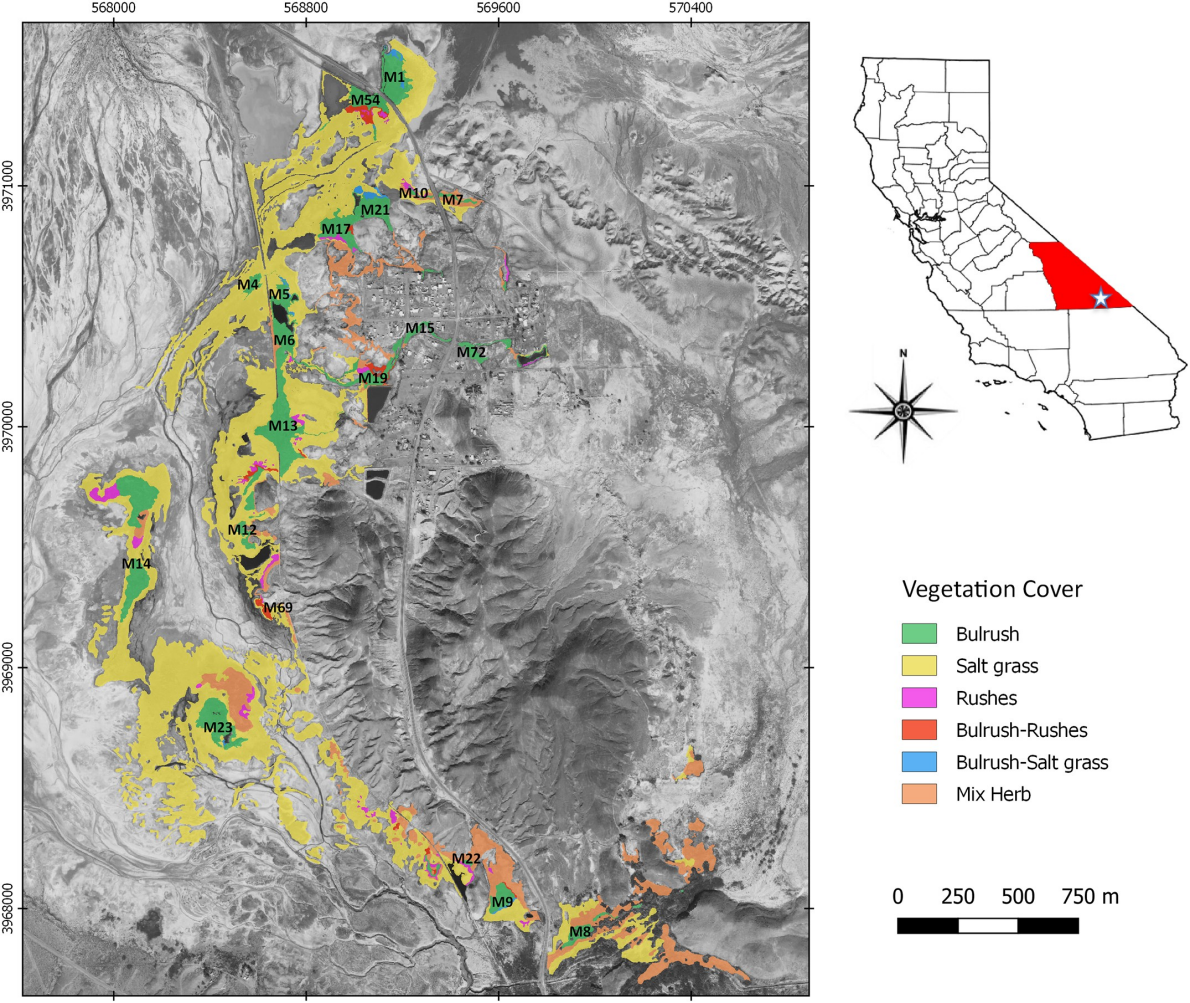
Inset map of Inyo County, California and detail of the Tecopa region showing locations of marshes and vegetation cover. Base map from Geological Society of America.

This study was conducted within the Amargosa River basin in the Mojave Desert near Tecopa, California (35.8752 N, -116.2343 E) at elevations from 390–417 m. Local climate is highly variable, with annual mean rainfall of 12.3 cm, and mean temperature ranges from 41.4 °C in summer to 3.2 °C in winter. Most of the Amargosa River in this region is subterranean, but ephemeral and spring-fed perennial surface flows in the region support marsh habitat. Marsh vegetation predominantly consists of bulrush (*Schoenoplectus americanus*) interspersed with a mix of desert salt grass (*Distichlis spicata*), rushes (*Juncus cooperi* and *J*. *balticus*), and mixed herb communities, including yerba mansa (*Anemopsis califcornica*), sunflower (*Helianthus annuus*), and western reed (*Phragmites australis*, Fig 1). We captured and translocated voles during summer 2014 and spring 2016. The 2014 work aimed to pilot use of radio-collars on Amargosa voles and determine survival rates and movement patterns of translocated voles vs. non-translocated voles. The 2016 work focused on the survival and movement patterns of translocated voles during a systematic translocation program of radio-collared voles into a sparsely inhabited marsh.

Initially, 12 marshes near Tecopa were evaluated to identify those with sustainable vole subpopulations (donor marshes) and to evaluate suitability of recipient marshes [16, 21]. In 2013, only Marsh 1 in the northeast corner of the study area appeared to have a sustainable subpopulation of voles based on trapping studies and population demographic analyses [22]. However, anthropogenically mediated impacts to Marsh 1 hydrology caused a significant decline in water level and reduction of bulrush density [15] such that in 2014, voles at Marsh 1 exceeded carrying capacity with survival rates of only 0.35 per month (R Klinger, USGS, and Foley, unpub. data) [23]. Thus Marsh 1 was chosen as a donor site for all 2014 and 2016 translocation recipient marshes. We designated candidate recipient marshes as those with at least 1 ha of mature bulrush, with standing water year-round, at least 0.5 km away from Marsh 1 to reduce risk of immediate return to the donor site, with no non-native predatory bullfrogs (*Lithobates catesbeianus*) [24], and housecats, and with very low or no vole subpopulations. We chose Marsh 9 as a recipient marsh in 2014, and Marshes 7, 8a, 19, 22, and 69 in 2016 (Fig 1).

### Trapping, translocation, and release of radio-collared voles

Marsh 1 was evaluated in summer 2014 and spring 2016 with six days of baseline trapping along pre-established grids of 108 large Sherman live traps (7.6 cm x 8.9 cm x 22.9 cm; HB Sherman, Tallahassee, FL). Recipient marshes were evaluated at least once in the three months pre-release during 2–4 day trapping periods along trap transects. Trap numbers varied by size of habitat fragment ranging from 30 to 44 traps per transect. Trap bait included either peanut butter, horse feed (corn, barley, oats, and wheat with molasses), and apples; or peanut butter and oats. Traps baited were placed under vegetation, kept open overnight, and then checked before dawn. Animals were examined for parasites, weighed, identified to sex, and marked with a uniquely numbered metal ear tag (1005–1 Monel, National Band and Tag Co., Newport, KY). Voles were assigned a body condition score (BCS) from 0–5 (0 is emaciated and 5 is obese) [25].

In 2014, we trapped, radio-collared, and monitored 10 voles: two male and two female voles were translocated from Marsh 1 to Marsh 9, three voles (1 male and 2 females) were captured and retained at Marsh 1 as donor marsh controls, and three voles (1 male and 2 females) were captured and retained at Marsh 9 as recipient marsh controls. In 2016, we randomly selected 20 healthy voles (9 males and 11 females) for collaring from among all voles captured in Marsh 1 for translocating to the five recipient marshes.

All 30 chosen voles had a BCS > 1+ and none was infested with chiggers. These voles were lightly anesthetized with 0.5 mg/kg ketamine and 0.1 mg/ml xylazine IM, received a passive integrated transponder (PIT) tag subcutaneously between the scapulae, and were fitted with a radio-transmitter on a zip-tie collar (total collar weight 2g; Biotrack Pip Ag392, Wareham, Dorset, UK). The collars had an expected battery life of 8 weeks, external antennae, a 33 ms pulse rate, and activity sensors. Based on numerous direct observations of collared voles and comparing locations indicated by the collars, collar location error was typically less than approximately a meter. Control voles were released at their capture site once completely recovered from anesthesia.

Voles selected for translocation were transported to the recipient marshes in Sherman traps and then transferred to acclimation cages (53 x 38 x 41 cm wire cages with 30 cm deep bulrush substrate, horse grain mixture, fresh bulrush, apples, lettuce, and water embedded on the margin of the recipient marsh) within approximately 2 hr. After 24 hr, one side of the acclimation cage was opened to allow the animal to leave as it chose. We provided food in the cage for one additional day. Before and after cages were opened, activity was monitored using remote cameras (Reconyx PC 900 Hyperfire, Holmen, WI) pointed at the acclimation cage openings.

### Radio-telemetry data collection

Radio-telemetry was performed using an R410 receiver (Advanced Telemetry Systems, Isanti, MN) for two months at each work period. In 2014, location data were collected by direct observation after walking through habitat to determine the exact location of each radio-collared vole. In 2016, in order to reduce disturbance of animals and damage to habitat, animal locations were estimated by triangulation, taking bearings on the radio signal from three different locations. Locations were visualized and analyzed with ArcMap 10.2 (ESRI, Redlands, CA). Minimum time alive (MTA) after radio-collaring voles was derived from the last recorded telemetry signals, recaptures of individuals, or findings of collars associated with a predation event. Animal locations were triangulated from the three bearings using the Mean Center tool. We estimated the area occupied (AO) by each vole by calculating the minimum convex polygon (MCP) with the Minimum Bounding Geometry tool. Maximum distance moved (MDM), which is the same as observed range length [26], was calculated for each vole as the maximum distance among all possible distances between location points using the Measure tool.

### Documenting habitat selection

We calculated the proportion of the different habitat types available in the study area based on a vegetation class layer developed using the Normalized Difference Vegetation Index (NDVI) from satellite imagery as part of a previous study [27]. Each vole location record was plotted in the vegetation cover layer, then habitat type was classified as the predominant vegetation within a 1 x 1m raster radius of each point location. Six habitat types were identified in the region: bulrush, salt grass, rushes, mixed bulrush/rushes, mix bulrush/salt grass, and mixed herbs.

### Statistical analysis

Three ANOVA models were performed to evaluate MTA after radio-collaring, AO, and MDM for resident vs. translocated voles (treatment), males vs. females, and 2014 vs. 2016, including an interaction term between sex and treatment. Data were analyzed with the statistical program “R” (R-Development Core Team, 2015) with P ≤ 0.05 used as a cutoff to infer statistical significance.

Assessment of habitat was performed by calculating the Manly´s standardized resource selection ratio (wi), which is the proportion of habitat used divided by the proportional availability of each habitat type. We pooled all individuals of the two work periods and measured habitat selection at the population level according to the study design type I described by Manly et al. (2002). We tested the selection ratios for Amargosa vole in each habitat using a chi-square test adjusted by Bonferroni confidence intervals. Habitat selection analysis was performed using package “adehabitatHS” [28] in R.

## Results

### Survival duration

Of the 24 translocated voles, seven (#s 674, 225, 470, 645, 128, 301, 288) were known to have survived from 74–202 days, while the others survived at least 4–49 days (Table 1). MTA did not differ between the two work periods, sex, or treatment (Table 2). Mean MTA of resident control voles was 38.2 days compared with 47.0 days for translocated voles and male survival was 31.5 days vs. 55.7 days for females (Table 2). Radio signals from two voles in Marsh 9 (307, a translocated animal and 638, a resident) were eventually found considerably north of the marsh in a patch of yerba mansa (*Anemopsis californica*) where a snake burrow was observed. Two California king snakes (*Lampropeltis getula* subsp. *californiae*) were observed by personnel in the marsh and captured on camera at the release cages. We suspect predation as a cause for the longer distance movement of these transmitters. One vole translocated to Marsh 22 (# 628) was found dead, with indications that the cause of death was a bird of prey.

**Table 1:** Activities of radio-collared translocated and non-translocated (control) Amargosa voles in seven different marshes. Treatments: DC = donor marsh control; RC = recipient marsh control; and T = Translocated voles. A indicates adult animal, SA is subadult; Body masses and body condition scores (BCS) are those at first and last capture, if available. MTA = minimum time alive; AO = area occupied as determined by minimum convex polygon; MDM = maximum distance moved.

Work period	ID	Treatment	Marsh	Sex	Age	BCS	MTA (days)	AO (ha)	MDM (m)
2014	196	DC	1	M	A	3, 2	14	0.0134	33.67
	558	DC	1	F	A	3, 2	43	0.3067	120.61
	601	DC	1	F	A	2, 3	132	0.0692	29.54
	220	RC	9	F	A	2+, na	13	0.2223	98.52
	360	RC	9	F	A	3, na	11	0.0763	47.13
	638	RC	9	M	A	3, 2+	16	0.2199	119.69
	307	T	9	F	A	2+, na	14	0.0461	88.23
	478	T	9	M	SA	2+, na	14	0.1746	88.73
	674	T	9	F	A	2, 2+	131	0.0004	53.73
	777	T	9	M	SA	2, na	5	0.0006	3.43
2016	018	T	7	F	A	2-, 2	22	0.2052	163.05
	225*	T	7	M	A	2, 2	99	0.0407	36.48
	271	T	7	M	A	3, na	10	0.3763	88.52
	470	T	7	F	A	2, 2-	202	1.4656	345.47
	111	T	8a	M	A	2, na	43	0.1861	76.65
	249	T	8a	M	A	3, na	10	0.0211	32.18
	512	T	8a	F	A	2, na	42	0.0795	61.22
	588	T	8a	F	A	2-, na	6	NA	69.34
	645	T	8a	F	A	2-, na	112	0.1805	58.57
	128	T	19	M	A	2-, na	91	0.2253	83.14
	212	T	19	M	A	2+, na	4	0.1278	58.91
	455	T	19	F	A	2, na	21	0.1316	50.26
	568	T	19	F	A	2, na	7	0.3866	170.88
	668	T	19	M	A	3, na	22	0.2751	65.55
	361	T	22	F	A	2, na	20	0.0307	29.37
	628	T	22	M	A	2, na	8	0.0168	26.15
	159	T	69	F	A	2-, na	49	0.0387	36.64
	225*	T	69	M	A	2, na	NA	0.0064	15.61
	301	T	69	F	A	2-, 2-	113	0.0364	51.16
	432	T	69	F	A	2, na	9	0.4774	199.81
	225*	T	1	M	A	2, na	NA	0.0598	76.03
	288	T	1	M	A	2+, na	74	0.0759	37.61

*This vole was relocated a second time after it returned it to the donor marsh (M1)

**Table 2:** Summary and ANOVA test statistics for the outcomes of minimum time alive (MTA), area occupied as determined by minimum complex polygon (AO), and maximum distance moved (MDM) among male and female Amargosa voles that were translocated among marshes in Tecopa California or left in their marshes as control (treatment) in 2014 and 2016.

Source of Variation	Level	Mean ± SE	d.f.	F	P
**Distance (MDM)**					
Year studied			1	0.342	0.563
	2014	68.3 ± 12.8			
	2016	83.3 ± 24.0			
Sex			1	3.368	0.077
	Female	98.4 ± 19.9			
	Male	57.8 ± 9.1			
Treatment			1	0.160	0.692
	Resident	74.9 ± 17.5			
	Translocated	82.3 ± 14.6			
Sex*Treatment			1	0.812	0.375
Residuals			25		
**Area (AO)**					
Year studied			1	0.508	0.482
	2014	0.11 ± 0.04			
	2016	0.15 ± 0.03			
Sex			1	0.429	0.518
	Female	0.15 ± 0.04			
	Male	0.12 ± 0.03			
Treatment			1	0.090	0.766
	Resident	0.15 ± 0.05			
	Translocated	0.13 ± 0.03			
Sex*Treatment			1	0.045	0.834
Residuals			25		
**Survival (MTA)**					
Year studied			1	0.201	0.658
	2014	39.3 ± 15.7			
	2016	48.2 ± 16.5			
Sex			1	1.621	0.214
	Female	55.7 ± 14.3			
	Male	31.5 ± 9.4			
Treatment			1	0.257	0.617
	Resident	38.2 ± 19.4			
	Translocated	47.0 ± 10.6			
Sex*Treatment			1	0.057	0.814
Residuals			25		

### Distance moved and area occupied

**Fig 2:**
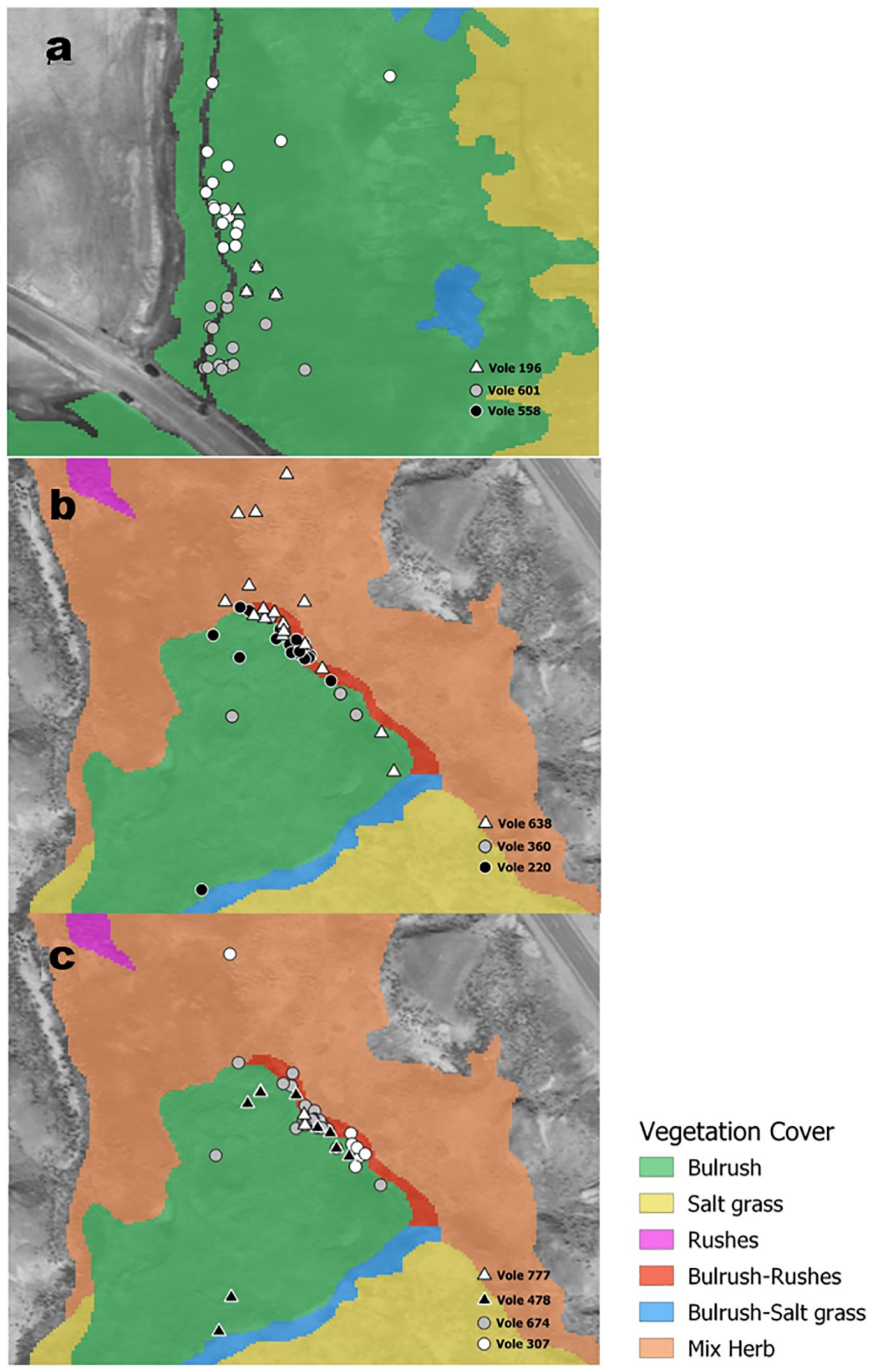
Map of radio-signals locations in different vegetation cover from three Amargosa voles that were residents at donor Marsh 1 (A), three residents at recipient Marsh 9 (B) and four translocated from Marsh 1 to Marsh 9 (C), near Tecopa CA in 2014. Triangles indicate males and circles indicate females.

**Fig 3:**
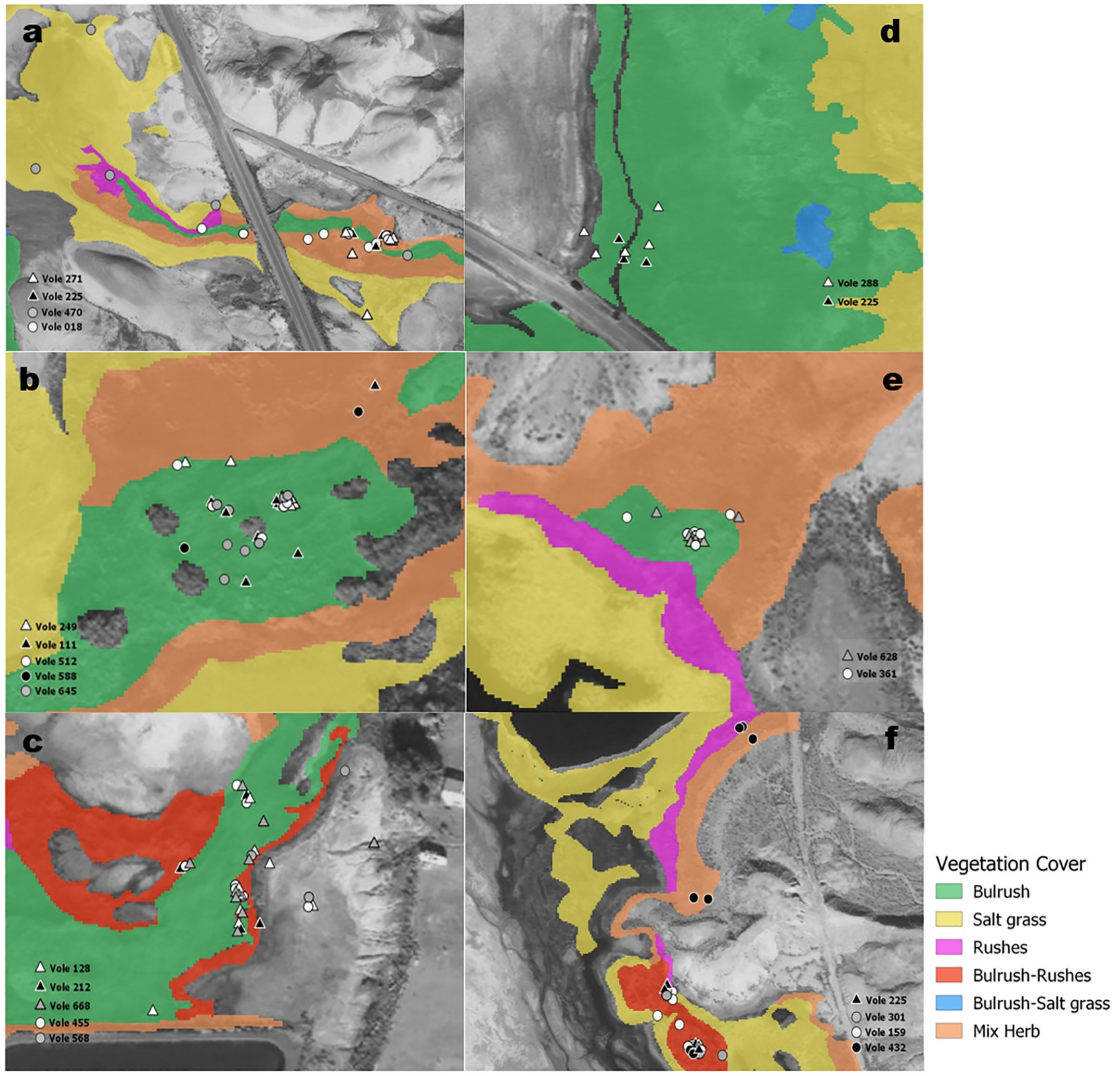
Map of radio-signals locations associated to vegetation cover from 20 Amargosa voles that were translocated at six recipient Marshes (A = Marsh 7,10; B = Marsh 8; C = Marsh 19; D = Marsh 1; E = Marsh 22; F = Marsh 69), near Tecopa CA in 2016. Triangles indicate males and circles indicate females.

Camera images showed that voles first exited acclimatization cages within 1.75–4 hrs after doors were opened and typically returned back to the cage several times over the next 2–3 days. In all cases, voles tended to be near to the cages during heat of the day while longer distance movements occurred at dawn, dusk, and night. The overall MDM average varied from 68.3 m in 2014 to 83.3 m in 2016 and most voles remained within 100 meters of the initial release site (Table 1, Figs 2 and 3). Five translocated voles left the boundaries of their recipient marshes (# 018, 225, 432, 470, and 518), three of which (# 225, 432, and 470) moved particularly far from release sites (200–577 m) (Table 1). Vole #225 moved from its recipient marsh (M7) and returned back to the donor marsh (M1) (Figs 1 and 3). Among the translocated voles who remained within their release marsh, five voles moved less than 30 m in any direction from their acclimation cage, and the remaining voles had MDM from 30–120 m. The mean MDM was greater for female vs. male voles and greater for control vs. translocated voles. None of the differences were statistically significant (Table 2).

For comparisons of AO, vole #470 was excluded from analyses due to limited data and because all recorded points indicated that this vole was traveling among marshes and had not settled. Points for vole #225, who had resided in three different marshes, were included if they were within the marsh but excluded when the vole was traveling among marshes. Overall the mean AO tended to be slightly higher for female vs. male voles and higher for resident control voles compared with translocated voles (Table 2). There were no significant differences in mean AO between 2014 and 2016, sex, or treatment (Table 2). Often, individual vole movement patterns showed overlap with other voles regardless of sex.

### Habitat selection

We analyzed 382 locations recorded from 30 Amargosa voles and the proportion of available habitat to evaluate vole habitat use (Table 3). Bulrush/rushes mix and bulrush were selected habitat, with the former approximately seven times more likely to be selected than the latter (B = 0.797 vs. B = 0.139). Although resident and translocated voles often moved through salt grass and rush patches, these two habitat types were significantly less likely to be used by voles than expected by chanc. We recorded some incidences of use of mixed herb habitat but this was not statistically significant (P = 0.74). There were no differences of habitat use preference and avoidance patterns of voles between the two work periods.

**Table 3:** Manly’s selectivity index (Wi) for each habitat use type by Amargosa voles in Tecopa, California. An index of ≥1 indicates that the habitat is used according to availability, while an index of <1 indicates avoidance.

Habitat	Proportion of habitat used	Proportion of habitat available	Wi + SE	Bi
Bulrush	0.613	0.149	4.12 ± 0.17*	0.139
Salt grass	0.029	0.720	0.04 + 0.01*	0.000
Rushes	0.016	0.020	0.80 +0.33	0.027
Bulrush-Rushes	0.241	0.010	23.61 + 2.15*	0.797
Bulrush-Salt grass	0.000	0.005	0.00 + 0.00*	0.000
Mix Herb	0.102	0.097	1.05 + 0.16	0.036

Wi = Selection ratios; SE = Error standard; Bi = Probability of habitat selection according to Manly’s standardized selectivity measure;

* significant values were based on Bonferroni level P > 0.0083

## Discussion

We describe a successful program of translocation of one of the rarest vertebrates in North America, and a species with some of the most restricted habitat requirements [10–12]. We also provide valuable new data on movement and space use of Amargosa voles, influences of sex on these behaviors, and support for the obligate dependence of the vole on bulrush marshes.

Our data clearly supported the selection of Amargosa voles for bulrush and mixed bulrush habitat, regardless of whether a vole was native to a marsh or had been translocated, with minimal use of salt grass and yerba mansa. Animals’ habitat selection reflects a balance in requirements for protection, nutrition, and opportunities to reproduce [29–31]. The bulrush and mixed bulrush habitat provides a major source of nutrition: Amargosa voles mostly consume seeds, leaves, shoots, culms and rhizomes of bulrush although we have incidental records of Amargosa voles feeding on yerba mansa and clustered goldenweed (*Pyrrocoma racemosa* var. *paniculata*) [12, 32]. In arid regions, small mammal populations are typically regulated by food availability determined by rainfall and subsequent primary production [33, 34], which might be happening in the Amargosa area wetlands. Amargosa voles in dense bulrush stands also receive protection against aerial predators via the overhead bulrush canopy and from pursuit predators by digging into bulrush litter. This litter also provides a thermal refuge against high summer temperatures and freezing winters, and a place to nest and rear young [11, 12, 27, 35]. Interestingly, our voles typically selected *mixed* patches with bulrush and other plant species over pure bulrush, in contrast with earlier work [12]. We acknowledge that some of the difference may be sampling effort, but earlier studies typically monitored voles only by where they were successfully trapped, whereas we followed them by radio-collar, which gives us greater insight into daily activity and habitat preferences. Amargosa voles likely have wider feeding niche breadth and move greater distances than previously reported, possibly to seek forage with higher fat and protein content than may be found in bulrush. Even though they appeared to avoid salt grass and rush patches, they must often cross through these areas when moving among marshes.

Amargosa voles in the present study include controls which presumably exhibited relatively normal behavior as well as translocated voles; these latter voles may remain in small areas until they become familiar with an area, adjust and exhibit normal behaviors an territory patroling, or leave the area altogether. Our “soft release” method was a variant of a release method described for water voles (*Arvicola amphibius*) [36]. Water vole cages had no bottoms and animals were able to leave cages by digging out. Amargosa vole release cages were opened after a day and these cages served as home base for the voles which typically returned multiple times to their cages. Studies in other rodents have reported difficulties with survival and animals remaining at the release sites, such as during translocation of prairie dogs (*Cynomys* spp.) after which only 0–40% showed site fidelity and ability to escape predation [37, 38]. Up to 100% of translocated prairie dogs dispersed if a starter cage was not provided, but if they were held for 5–15 days in a cage, almost all animals remained at the site [39]. In this context, the survival rates for translocated Amargosa voles and willingness of many voles to remain at the release site represent excellent outcomes.

Despite that Amargosa voles seem obligately dependent on marsh habitat, occasional extensive travel was detected, including five voles that left their recipient marshes, two moving from 300–500 m between different marshes. In contrast, translocated collared short-tailed voles (*M*. *agrestis*) and bank voles (*Clethrionomys glareolus*), but not meadow voles (*M*. *pennsylvanicus*), appeared less likely to move compared with non-translocated controls [40, 41]. Spacing behavior, social interactions, and territoriality all influence California vole demography [42]. Amargosa voles may leave a release marsh because of intra-guild aggression as we have seen previously on camera traps [32], seeking a territory, disorientation, or other unknown causes.

Due to relatively small numbers of locations obtained for each individual, we used MCP to estimate each vole’s AO as is described in the literature [43–45]. Excluding five translocated voles that left their recipient marshes, the AO by Amargosa voles was consistent with several other vole species including *M*. *agrestis* [46], *M*. *oeconomus* [47], and *Arvicola sapidus* [48], in which a range of 0.003–0.286 ha was reported. Although area occupied by small mammals may be affected by resource availability, density, breeding season, and sex [46, 49], we did not find any statistical differences between males and females. There was also no difference between resident and translocated voles. The observations of voles commonly using areas also used by other voles regardless of sex suggest some tolerance by Amargosa voles for other animals of the same and opposite sex, although this behavior should be further examined with a larger sample size.

Vole mortality post-release is an important consideration for Amargosa vole translocation actions. Similar to previous studies [12, 50], voles that were captured and recaptured in the present study usually had good body condition without evidence of starvation or disease. Given that radio-telemetry signal times were only live from 1–43 days and the recapture rate was low, we were not able to confirm the survival rate of all voles and our MTA are underestimates. The high MTA up to 202 days and the finding that average MTA was did not differ between translocated and resident voles support that many voles successfully evaded predation after translocation, even though natural predators were present in all marshes and some voles were documented to succumb to predation. For translocated prairie dogs, availability of a well-developed extant or human-constructed burrow system was crucial for protection against predators [37]. The average lifespan of an Amargosa vole in the wild is limited to only a few months due to intense predation [17, 21, 51], even though Amargosa voles in captivity may live for several years [52]. Individuals escape predation by tunneling in deep bulrush litter and sometimes soil, whereas populations persist by having large numbers of expendable offspring (an r-selection life history strategy). Additional research on predation may be needed to support management of heavily impacted subpopulations.

Expected challenges in this study were low survival, emigration, or ultimately unsuccessful reproduction. Although we didn’t attempt translocation during all seasons, success during summer and spring seasons suggests seasonal adequacy of food resources and that this is an appropriate time for such action. Nevertheless, further studies providing a more robust data set are warranted. As anthropogenic challenges to wildlife habitat accumulate and more animals—particularly those that are highly endemic and specialized within their ecosystems—become increasingly management-dependent, having high-quality and well-studied protocols for intervention to stabilize subpopulations becomes critically necessary. Translocation is an attractive option for intensive endangered species management [53–56], and yet translocated territorial animals face risks involved in establishing new territories, finding food, and escaping local threats. Indeed, critics suggest that translocation is unnatural, may be cruel, may fail to yield sustainable populations, may promote disease spread, and may fail to protect against future habitat changes [57–62].

However, with the Amargosa vole’s distribution so strongly influenced by availability of water and bulrush [11], scarcer and less predictable water in the Mojave Desert will require trustee agencies to implement intensive intervention through management of habitat as well as direct support for animal populations. Larger founder populations, high quality habitat, and release into core of habitat increase the probability of translocation success [63–65]. Even when a single large translocation is not possible, multiple smaller augmentation events may be even more useful to keep populations from succumbing to Allee effects [57, 66]. Furthermore, data gathered from translocation studies are useful to inform habitat management planning to support endangered species. The scale of the intervention required to create sustainable populations of such an r-selected and highly endemic species is modest. With suitable incorporation of safeguards against transmitting disease, translocation is a useful tool to support a healthy metapopulation of Amargosa voles for years to come.
